# Electrocardiogram Classification Based on Faster Regions with Convolutional Neural Network

**DOI:** 10.3390/s19112558

**Published:** 2019-06-05

**Authors:** Yinsheng Ji, Sen Zhang, Wendong Xiao

**Affiliations:** 1 School of Automation and Electrical Engineering, University of Science and Technology Beijing, Beijing 100083, China; s20180575@xs.ustb.edu.cn (Y.J.); zhangsen@ustb.edu.cn (S.Z.); 2 Key Laboratory of Knowledge Automation for Industrial Processes of Ministry of Education, School of Automation and Electrical Engineering, University of Science and Technology Beijing, Beijing 100083, China

**Keywords:** electrocardiogram, electrocardiogram preconditioning, deep learning, convolutional neural network, automatic classification

## Abstract

The classification of electrocardiograms (ECG) plays an important role in the clinical diagnosis of heart disease. This paper proposes an effective system development and implementation for ECG classification based on faster regions with a convolutional neural network (Faster R-CNN) algorithm. The original one-dimensional ECG signals contain the preprocessed patient ECG signals and some ECG recordings from the MIT-BIH database in this experiment. Each ECG beat of one-dimensional ECG signals was transformed into a two-dimensional image for experimental training sets and test sets. As a result, we classified the ECG beats into five categories with an average accuracy of 99.21%. In addition, we did a comparative experiment using the one versus rest support vector machine (OVR SVM) algorithm, and the classification accuracy of the proposed Faster R-CNN was shown to be 2.59% higher.

## 1. Introduction

An electrocardiogram (ECG) as a cardiac activity record provides important information about the state of the heart [1]. ECG arrhythmia detection is necessary for early diagnosis of heart disease patients. On the one hand, it is very difficult for a doctor to analyze an electrocardiogram with a long recording time for a limited time. On the other hand, people are also almost unable to recognize the morphological changes of ECG signals without tool support. Therefore, an effective computer-aided diagnosis system is needed to solve this problem.

Most ECG classification methods are mainly based on one-dimensional ECG data. These methods usually need to extract the waveform’s characteristics, the interval of adjacent wave, and the amplitude and period of each wave as input. The main difference between them is the selection of the classifier.

Early on, Yuzhen et al. [2] used the BP neural network to classify the ECG beat, with the classification accuracy rate reaching 93.9%. Martis et al. [3,4] proposed discrete cosine transform (DCT) coefficients from the segmented beats of ECGs, which were then subjected to principal component analysis for dimensionality reduction, and a probabilistic neural network (PNN) for automatic classification. They classified the ECG beats into five categories with the highest average sensitivity of 98.69%, specificity of 99.91%, and classification accuracy of 99.52%. Luo et al. used an artificial neural network, based on multi-order feedforward, to classify the ECG beat into six categories, and finally obtained a classification accuracy rate of 90.6% [5]. Osowski et al. designed a classifier that cascades the fuzzy self-organizing layer and the multi-layer perceptron, and realized seven classifications for ECG beats with a classification accuracy rate of 96% [6]. Ceylan et al. used feedforward neural networks as the classifier, and they realized the detection of four different arrhythmias with an average accuracy of 96.95% [7]. Hu extracted features based on multiple discriminant and principal component analysis, and used a support vector machine (SVM) for classification [8]. Song et al. used a combination of linear discriminant analysis with SVM for six types of arrhythmia [9]. Melgani and Bazi proposed SVM with particle swarm optimization for the classifier. Finally, they achieved 89.72% overall accuracy with six different arrhythmias [10]. Martis et al. [11] used a four-layered feedforward neural network and a least squares support vector machine (LS-SVM) to classify the ECG beats into five categories with highest average accuracy of 93.48%, average sensitivity of 99.27%, and specificity of 98.31%. Pławiak [12] designed two genetic ensembles of classifiers optimized by classes and by sets, based on an SVM classifier, a 10-fold cross-validation method, layered learning, genetic selection of features, genetic optimization of classifiers parameters, and novel genetic training. The best genetic ensemble of classifiers optimized by sets obtained a classification sensitivity of 17 heart disorders (classes) at a level of 91.40%.

Deep learning was successfully applied to many areas such as recognizing numbers and characters, face recognition, object recognition, and image classification. Deep learning methods are also used effectively in the analysis of bioinformatics signals [13,14,15,16]. Ubeyli proposed a recurrent neural network (RNN) classifier with an eigenvector-based feature extraction method [17]. As a result, the model achieved 98.06% average accuracy with four different arrhythmias. Kumar and Kumaraswamy introduced a random forest tree (RFT) as the classifier which used only an RR interval as the classification feature [18]. Park et al. proposed a K-nearest neighbor (K-NN) classifier for detecting 17 types of ECG beats, which resulted in an average of 97.1% sensitivity and 98.9% specificity [19]. Jun et al. also introduced K-NN, proposing a parallel K-NN classifier for high-speed arrhythmia detection [20]. Related to this paper, Kiranyaz et al. introduced a one-dimensional (1D) convolutional neural network (CNN) for the ECG classification, where they used CNN to extract features for one-dimensional ECG signals [21]; however, the classification accuracy was not higher than our method. Rajpurkar et al. also proposed a one-dimensional CNN classifier that used deeper and more data than the CNN model of Kiranyaz [22]. Despite using a larger ECG dataset, the classification performance was still lower than our model. The reason is that, although the size of the dataset was increased, the ECG signal used as an input remained in one dimension; thus, the performance improvement was not great even with deep CNN [23]. Yildirim et al. [24] designed a new 1D convolutional neural network model and achieved a recognition overall accuracy of 17 cardiac arrhythmia disorders at a level of 91.33%. Acharya et al. [25] developed a nine-layer deep CNN to automatically identify five different heartbeat types. Their proposed model achieved 94.03% and 93.47% accuracy rates in the original and noise-free ECGs, respectively. Shu et al. [26] modified the U-net model to perform beat-wise analysis on heterogeneously segmented ECGs of variable lengths derived from the MIT-BIH arrhythmia database, and attained a high classification accuracy of 97.32% in diagnosing cardiac conditions. Chauhan and Vig [27] used deep long short-term memory (LSTMs) networks to detect abnormal and normal signals in ECG data. The ECG signals used included four different types of abnormal beats. The proposed deep LSTM-based detection system provided 96.45% accuracy on test data. Yildirim [28] proposed a new model for deep bidirectional LSTM network-based wavelet sequences, and classified the heartbeats obtained from the MIT-BIH arrhythmia database into five different types with a high recognition performance of 99.39%. Tan et al. [29] implemented LSTM with CNN to automatically diagnose coronary artery disease (CAD) using ECG signals accurately. Warrick and Homosi [30] proposed a new approach to automatically detect and classify cardiac arrhythmias in ECG records. They used a combination of CNN and LSTM. Shu et al. [31] also proposed an automated system using a combination of CNN and LSTM for diagnosis of normal sinus rhythm, left bundle branch block, right bundle branch block, atrial premature beats, and premature ventricular contraction on ECG signals. They achieved an accuracy of 98.10%, sensitivity of 97.50%, and specificity of 98.70%. Hwang et al. [32] proposed an optimal deep learning framework to analyze ECG signals for monitoring mental stress in humans.

In this paper, we propose an ECG classification method using faster regions with a convolutional neural network (Faster R-CNN) with ECG images. This method uses CNN to extract features of ECG images. The reason why we applied two-dimensional CNN by converting the ECG signal into an ECG image in this paper is that two-dimensional convolutional and pooling layers are more suitable for filtering the spatial locality of the ECG images. As a result, higher ECG classification accuracy can be obtained. In addition, the physician can judge the arrhythmia in ECG signals of the patient through vision treatment of the eyes. Therefore, we concluded that applying the two-dimensional CNN model to the ECG image is most similar to the physician’s arrhythmia diagnosis process. Moreover, this method can be applied to ECG signals from various ECG devices with different sampling rates. Before the one-dimensional ECG signals are converted to two-dimensional ECG images, we preprocessed the one-dimensional ECG signals by empirical mode decomposition (EMD) [33]. Finally, we classified the ECG into five categories with 99.21% average accuracy. Meanwhile, we did a comparative experiment using the OVR SVM algorithm, and the classification result of our method is higher than that of the one versus rest (OVR) SVM [34] algorithm. At the end of the article, we compared our model with previous works using machine learning algorithms to classify ECG, where the proposed method achieved the best results in average accuracy.

The rest of the paper is structured as follows: Section 2 introduces the method of ECG signal preprocessing and Faster R-CNN architecture. Section 3 presents the experimental design based on the Faster R-CNN algorithm. Section 4 describes the experimental results and the comparative analysis. Conclusions are drawn in Section 5.

## 2. Methods

**Figure 1:**
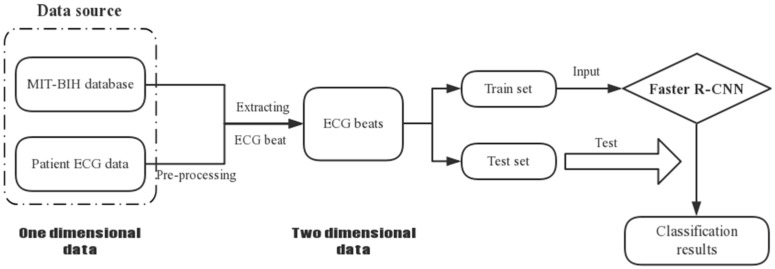
Overall procedure involved in electrocardiogram (ECG) beat classification.

In this paper, we used one-dimensional ECG data from the recordings of the MIT-BIH database and the patient. We firstly preprocessed the ECG data from the patient, which had quite serious noise interference. Since the Faster R-CNN model handles two-dimensional images as input data, we transformed the ECG signals into ECG images. Finally, we used Faster R-CNN to classify the ECG beats into five categories. The overall procedure is shown in Figure 1.

### 2.1. ECG Signal Pre-Processing

In general, due to the weakness of the ECG signal and the influence of acquisition equipment, many interference noises would be easily mixed during the acquisition process. However, these noises are very unfavorable for the analysis of ECG signals. Therefore, effective preprocessing of ECG signals is a key issue before the classification of ECG. Common ECG signal interference noises include power frequency interference, baseline drift, and myoelectric interference.

**Figure 2:**
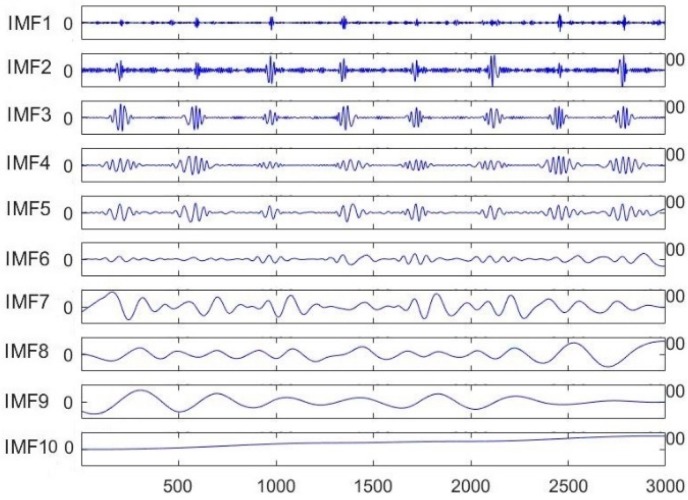
Individual intrinsic mode functions (IMFs) after ECG decomposition.

As shown in Figure 2, the original ECG signal from the patient was decomposed into 10 intrinsic mode functions (IMFs) using the EMD algorithm. Among them, the noise signal was mainly concentrated in the IMF1 and IMF2 modes. The baseline drift was mainly focused on the IMF9 and IMF 10 modes, while the remaining modes contained important information about the ECG signal.

**Figure 3:**
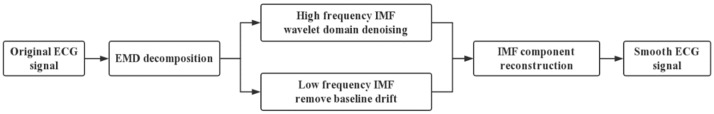
ECG signal processing flow based on empirical mode decomposition (EMD).

The high-frequency IMF (IMF1 and IMF2) modes were denoised by the wavelet transform algorithm, while the baseline drift of the low-frequency IMF (IMF9 and IMF10) modes was eliminated by the median filtering algorithm. The processed IMF modes and the remaining unprocessed modes were reconstructed, and a smooth and noiseless ECG signal was finally obtained. The process is shown in Figure 3.

**Figure 4:**
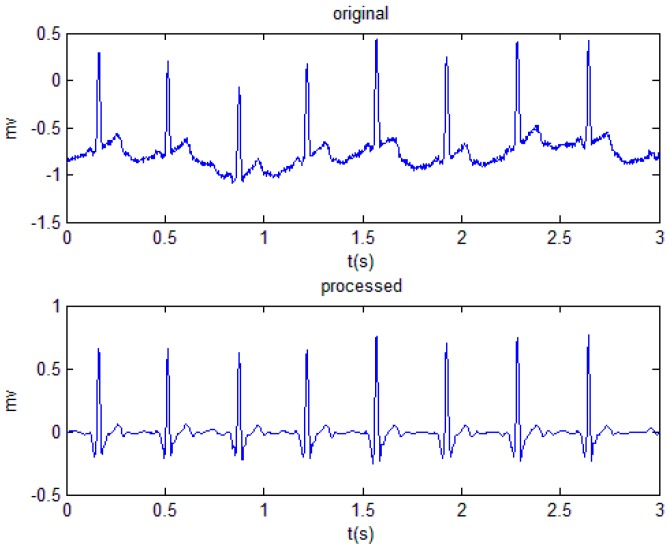
Comparison of the original signal and the processed signal.

As shown in Figure 4, the ECG signal without noise and baseline drift makes it easier to classify the ECG signal.

### 2.2. Transforming the ECG Signals into ECG Images

Before transforming the one-dimensional ECG signal into a two-dimensional image, the R wave position of the ECG signal needs to be found. In this paper, discrete wavelet transform (DWT) [35] was adopted to find the R wave.

#### 2.2.1. R Wave Detection

Wavelet transform achieved good results in improving the anti-interference and accuracy of the QRS group detection. According to the wavelet transform theory, the R wave peak point corresponds to the zero crossing of the modulus maxima. The R wave peak position is located by detecting the position of the modulus maxima of the R wave, and then the start and end points of the QRS wave are searched forward and backward according to the R peak position.

**Figure 5:**
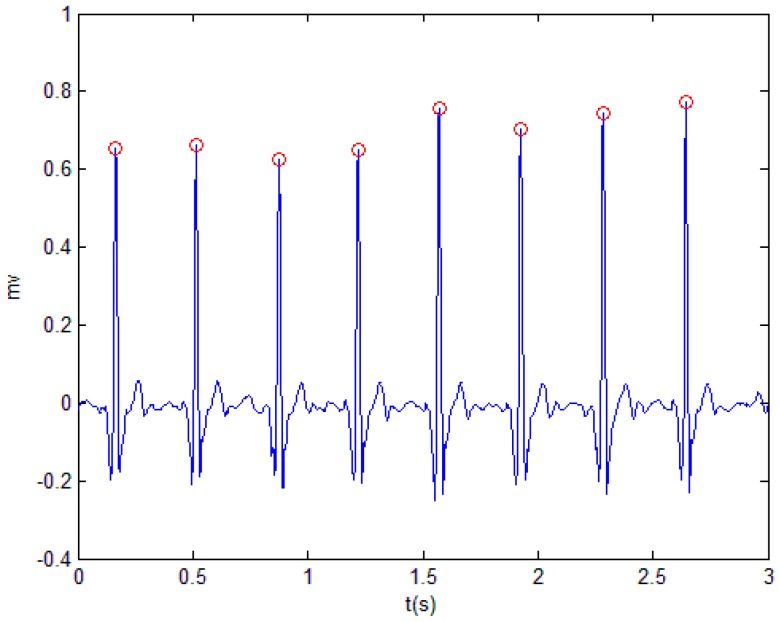
Find the position of the R wave based on discrete wavelet transform (DWT).

In the experiment, the DWT method and the adaptive threshold denoising method were used to detect the R wave of the ECG signal. The modulus maximum and the zero crossing were detected to find the position of the QRS group. Then, the adaptive noise threshold method was used to judge whether the detected peak position was an R wave or a glitch. Figure 5 shows the position of the R wave point in the ECG signal.

#### 2.2.2. Extracting the ECG Beat

**Figure 6:**
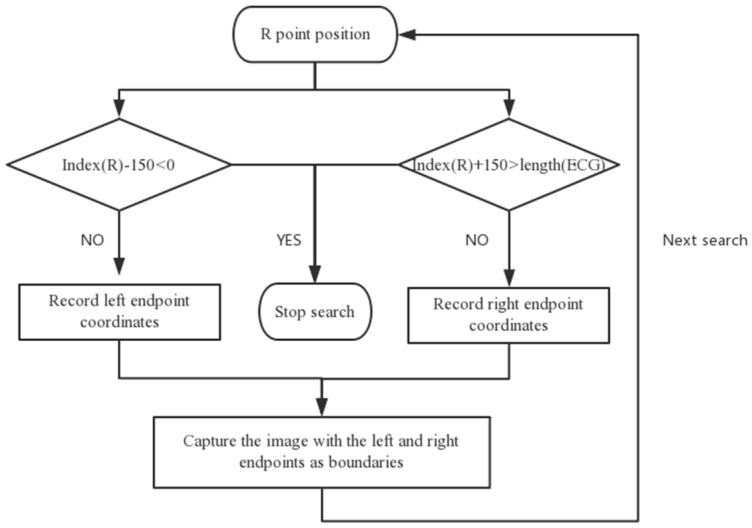
ECG beat extraction process.

**Figure 7:**
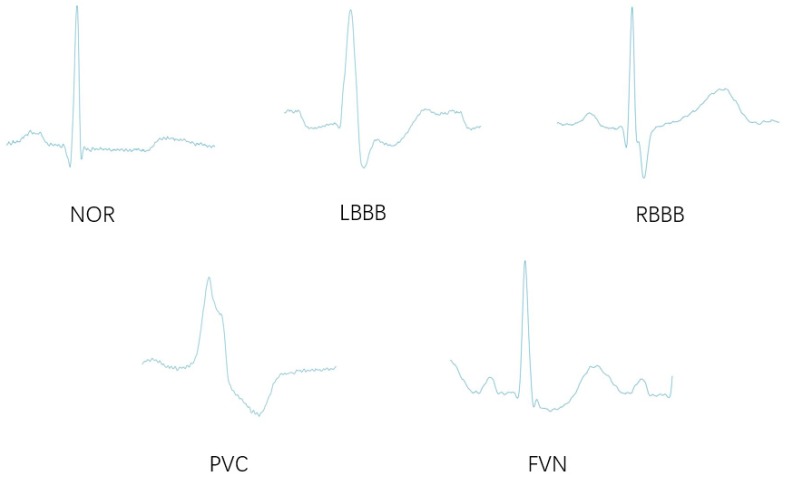
Normal beat and four ECG arrhythmia beats.

In this paper, the sliding window search method was used to extract the ECG beat. The current R wave point was used as a reference to search the left for 150 ms. If the point existed, the coordinates of the left endpoint were recorded; otherwise, the search was stopped. Then, the right was searched for 150 ms with the current R wave point as reference. If the location point existed, the coordinates of the right endpoint were recorded; otherwise, the search was stopped. Finally, we cut the graph from the left to the right endpoints as the input sample of the deep learning network in the experiment. Figure 6 shows the process of extracting the ECG beats, and Figure 7 shows the ECG beat samples.

### 2.3. Faster R-CNN Architecture

**Figure 8:**
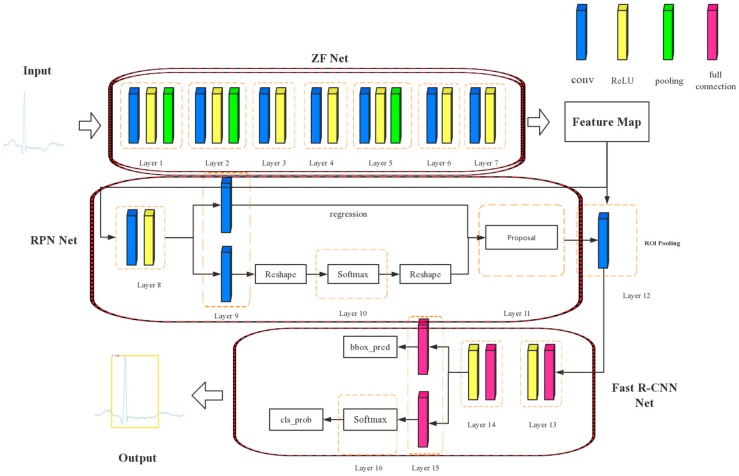
Faster regions with a convolutional neural network (Faster R-CNN) architecture.

In this paper, we used Faster R-CNN based on the ZF net to classify the ECG. As we can see in Figure 8, Faster R-CNN is composed of the ZF net, region proposal network (RPN) net, and Fast R-CNN net. Among them, the ZF net is a CNN architecture, which is used to extract the feature map of ECG images. Then, the RPN net runs on the feature map and generates approximately 20,000 rectangular boxes, which are sorted according to scores from large to small. Then, the first 300 rectangular boxes are taken as inputs for the Fast R-CNN net, which maintains higher accuracy while reducing time complexity. Finally, the Fast R-CNN net outputs the probability of a category and a coordinate matrix (containing four coordinate values).

#### 2.3.1. Region Proposal Network

The region proposal network adopts the neural network, and integrates the three processes of generating candidate boxes, extracting features, and classifying them into a network model. Finally, the RPN realizes end-to-end training and detection. The RPN takes an image of any size as input and outputs a set of candidate boxes, where each box has a score for evaluating the similarity between the box and the target.

The small network slides on the convolution feature map of the last layer output of the shared convolutional layer. Each sliding window is mapped to a low-dimensional vector, which is output to two fully connected layers of the same level—the rectangular frame regression layer and the rectangular frame classification layer.

**Figure 9:**
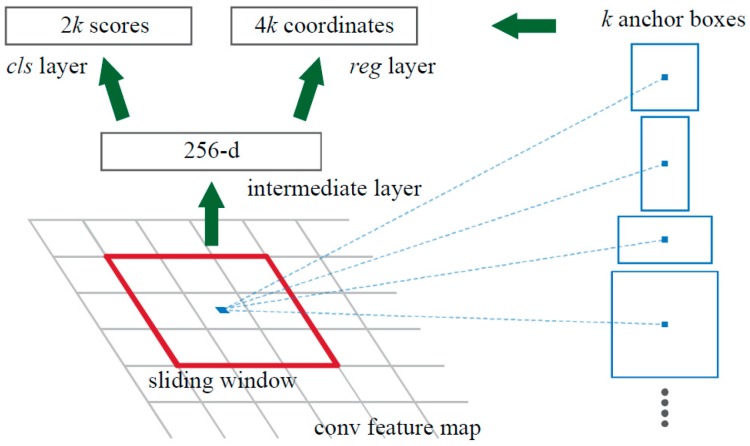
Region proposal network [36].

An anchor mechanism is proposed by Faster R-CNN. As shown in Figure 9, at each position of the sliding window, *k* area suggestions are predicted at the same time, so the rectangular frame regression layer has 4*k* outputs, that is, coordinate values of *k* boxes (*x*_1, *y*_1, *x*_2, *y*_2). The rectangular frame classification layer has 2*k* outputs. The center of the *k* anchors is the center of the current sliding window. Faster R-CNN uses three scales and three aspect ratios; thus, there are *k* = 9 anchors at each sliding position. There is a total of W × H × *k* anchors for a convolutional feature map of size W × H [36].

#### 2.3.2. Loss Function

Each anchor is assigned a label that is a “target” (positive label) or “non-target” (negative label). The Faster R-CNN network inherits the multitasking loss mechanism of the Fast R-CNN network. For an $anchor_{i}$, its loss function is defined as

(1) $$L\left(\left\{p_{i}\right\},\left\{t_{i}\right\}\right)=\frac{1}{N_{cls}}\sum_{i}L_{cls}\left(p_{i},p_{i}^{*}\right)+\lambda \frac{1}{N_{reg}}\sum_{i}p_{i}^{*}L_{reg}\left(t_{i},t_{i}^{*}\right),$$

where *i* is the index of an anchor in the mini-batch, and $p_{i}$ is the predicted probability of the condition that $anchor_{i}$ is a positive label. If the anchor is a positive label, $p_{i}$* is 1; otherwise, $p_{i}$* is 0. $t_{i}=\left\{t_{x},t_{y},t_{w},t_{h}\right\}$ is a vector representing the four parameterized coordinates of the predicted rectangular box, and $t_{i}^{*}=\left\{t_{x}^{*},t_{y}^{*},t_{w}^{*},t_{h}^{*}\right\}$ is the coordinate vector of the rectangle corresponding to the anchor with a positive label.

The classification loss function in Equation (1) is

(2) $$L_{cls}\left(p_{i},p_{i}^{*}\right)=-log\left[p_{i}p_{i}^{*}+\left(1-p_{i}\right)\left(1-p_{i}^{*}\right)\right].$$

The regression loss function in Equation (1) is

(3) $$L_{reg}\left(t_{i},t_{i}^{*}\right)=R\left(t_{i}-t_{i}^{*}\right).$$

The R function in Equation (3) is the smooth L1 function shown in Equation (4).

(4) $${\mathrm{smooth}}_{L1}\left(x\right)=\{\begin{array}{cc} 0.5x^{2}, & \mathrm{if}\;\left|x\right|<1\\ \left|x\right|-0.5, & \mathrm{otherwise} \end{array}.$$

The outputs of the rectangular box classification layer and the rectangular box regression layer are composed of {$p_{i}$} and {$t_{i}$}, respectively. They are normalized by the parameters $N_{cls}$, $N_{reg}$, and *λ*. The training process is developed around the loss function *L*({$p_{i}$}, {$t_{i}$}), toward the reducing direction of *L*({$p_{i}$}, {$t_{i}$}).

#### 2.3.3. Convergence Feature Sharing

Faster R-CNN developed a technology which allows the sharing of convolutional layers between the RPN network and Fast R-CNN network, and it achieves joint training rather than learning the two networks separately. This algorithm consists of four steps, the basic idea of which is to alternate optimization.

(a) Initialize the network parameters with a pre-trained model on ImageNet, and fine-tune the RPN network [37];
(b) Use the initialized RPN network in step (a) to extract the region proposal training Fast R-CNN network;
(c) Re-initialize the RPN using the Fast R-CNN network in step (b), fix its convolutional layer while training the RPN network, and only fine-tune its unique layer;
(d) Fix the convolutional layer parameters after the Fast R-CNN learning in step (b). On this basis, use the region proposal extracted by the RPN in step (c) to fine-tune the Fast R-CNN network.

#### 2.3.4. Non-Maximum Suppression

**Figure 10:**
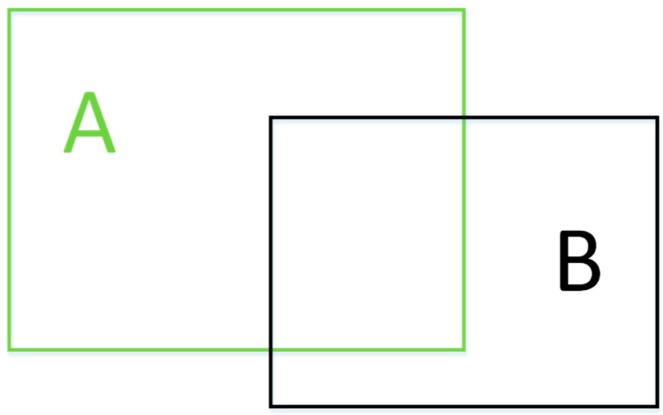
Intersection-over-union (IoU).

As shown in Figure 10, intersection-over-union (IoU) is used to measure the degree of overlap between the two rectangular boxes, which can be defined as the ratio of the intersection of the two boxes to the union.

(5) $$\mathrm{IoUA},B=\frac{A\cap B}{A\cup B}.$$

**Figure 11:**
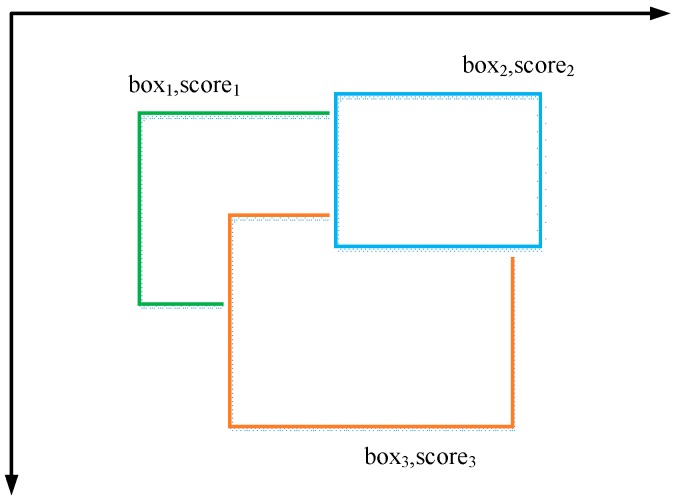
Non-maximum suppression.

Non-maximum suppression (NMS) is a strategy for finding maxima and suppressing non-maximal values according to certain rules. In target detection, NMS is often used to remove redundant windows. As shown in Figure 11, all boxes are sorted according to their score from small to large. The IoU between the rest of the box is calculated based on the box of the highest score. If the IoU exceeds the threshold set in advance, then the corresponding box is suppressed.

## 3. Experimental Design

### 3.1. Experimental Platform

This experiment was conducted under the Windows 10 operating system. Table 1 lists the software information in the experiment.

### 3.2. ECG Beat Classification Criteria

There are many types of ECG beats, and many similarly shaped beats must rely on physicians with specialized experience to be able to accurately identify them. Table 2 shows the currently accepted classification standard for ECG beats.

The classification performance is measured by four criteria, specificity (Spe), sensitivity (Sen), positive rate (Pre), and accuracy (Acc), as follows:

(1) Specificity (Spe): The proportion of normal ECG beats that are correctly classified, which represents the correct rate of non-patients.
(2) Sensitivity (Sen): The proportion of abnormal ECG beats that are correctly classified, which represents the correct ratio of patients.
(3) Positive rate (Pre): The number of abnormally classified ECG beats is correctly classified as the proportion of abnormal ECG beats.
(4) Accuracy (Acc): Proportion of correct classification of ECG beats to all ECG beats.

Table 3 shows the definition of the four classification results. According to the definitions of specificity, sensitivity, and accuracy, the formulas are as follows:

(6) $$Spe=\frac{TN}{TN+FP};$$

(7) $$Sen=\frac{TP}{TP+FN};$$

(8) $$Acc=\frac{TP+TN}{ALL\;NUM}.$$

### 3.3. Building Dataset

The ECG data used in this paper were obtained from the MIT-BIH database, and some patients cooperated with us. Among the data, the MIT-BIH database contained 48 half-hour ECG recordings collected from 47 patients. There are approximately 110,000 ECG beats in the MIT-BIH database with 15 different types. From the MIT-BIH database, we included normal beat (NOR) and four types of ECG arrhythmias, including left bundle branch block beat (LBBB), right bundle branch block beat (RBBB), premature ventricular contraction (PVC), and fusion of ventricular and normal beat (FVN). Table 4 shows the number of ECG beats per class in the MIT-BIH database.

To ensure the balance of the dataset, we extracted some NOR beats from the 18 records to make the NOR data set. The final number of NOR beats in the dataset was 8500. Because the number of FVN beats was too small, we extended the FVN beats by translation and flipping. The final number of FPN beats was 8030.

After getting the two-dimensional images, we renamed each ECG image to a six-digit file name (e.g., 000001.jpg), and adjusted the pixel value to 500 × 375 for Faster R-CNN input format. Then, we marked each ECG beat image manually and saved the information into an XML file. The file naming rules were consistent with the image, e.g., 000001.xml. The dataset is shown in Table 5.

### 3.4. Network Parameter Optimization

#### 3.4.1. Impact of Learning Rate on Classification Performance

Learning rate is an important parameter in deep learning. If the learning rate is too large, it is easy to overshoot the phenomenon. If the learning rate is too small, this will lead to slow convergence or overfitting. Therefore, it is necessary to consider the actual problem to determine the learning rate. Table 6 shows the effect of learning rate on the performance of this experimental classification.

As the learning rate decreases, the loss function value decreases and gradually stabilizes. Although the classification accuracy is the highest at the learning rate of 0.001, the training takes longer; thus, it was more appropriate to consider the learning rate as 0.001.

#### 3.4.2. Effect of Weight Attenuation Coefficient on Classification Performance

In the loss function, the weight decay is the coefficient placed before the regularization. Regular terms usually represent the complexity of the model. Therefore, the effect of changing the weight attenuation coefficient is to adjust the effect of the loss function. If the weight attenuation is greater, the value of the loss function is also larger. Table 7 shows the effect of the attenuation weighting factor on the performance of the experimental classification.

As the weight attenuation coefficient decreases, the loss function value decreases; the classification accuracy is the highest when the weight attenuation coefficient value is 0.0005. Therefore, considering the weight, the weight attenuation coefficient was set to 0.0005.

#### 3.4.3. Influence of the Number of Iterations on Classification Performance

Iterations represent the maximum number of iterations during the training process. If the parameter is too small, the model will not be adequately trained. If it is too large, the model may suffer from over-fitting. Table 8 shows the impact of the number of iterations on classification performance.

Based on the results in Table 8, the final number of iterations was chosen as 4000.

## 4. Experimental Results and Comparative Analysis

### 4.1. Experimental Results

**Figure 12:**
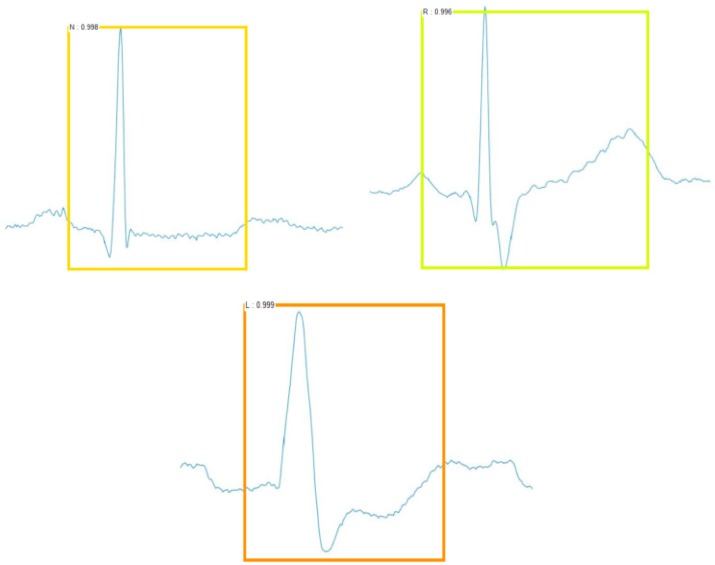
The detected ECG beats.

The Faster R-CNN framework used in this paper has three aspects for the classification of ECG beats: target position box, target classification, and score. The target position involves the coordinates of the upper left vertex and the lower right vertex of a rectangular box. The target classification involves the result of the model’s judgment on the image category at that position, which had only five discrete values in this experiment. The score is a probability value to show how likely the ECG beat is to be in this category, which is also called the confidence coefficient. The ECG beats were classified as shown in Figure 12.

Precision rate and recall rate are also two important evaluation indexes for classification results. According to the definition in Table 3, the formulas of precision rate and recall rate are as follows:

(9) $$Precision=\frac{TP}{TP+FP};$$

(10) $$Recall=\frac{TP}{TP+FN}.$$

Because the same ECG beat could be divided into multiple categories and each category had a score, we needed to set a score threshold to remove the categories with low scores. However, when the score threshold is too low, multiple target frames will appear, and the precision rate will decrease. When the score threshold is too high, the precision rate will increase, and the recall rate will decrease.

**Figure 13:**
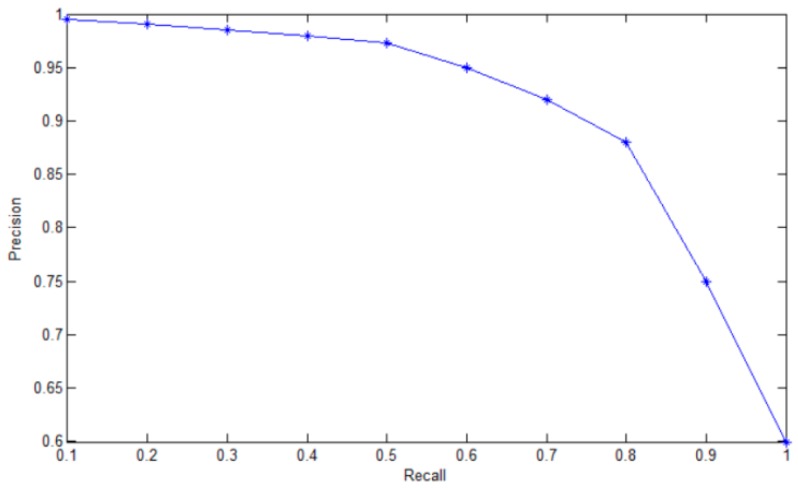
Score threshold test results.

Figure 13 plots the relationship between precision rate and recall rate when we adjusted the score threshold. After many experiments, when the score threshold value was equal to 0.7, the precision rate and the recall rate could reach a better level.

**Figure 14:**
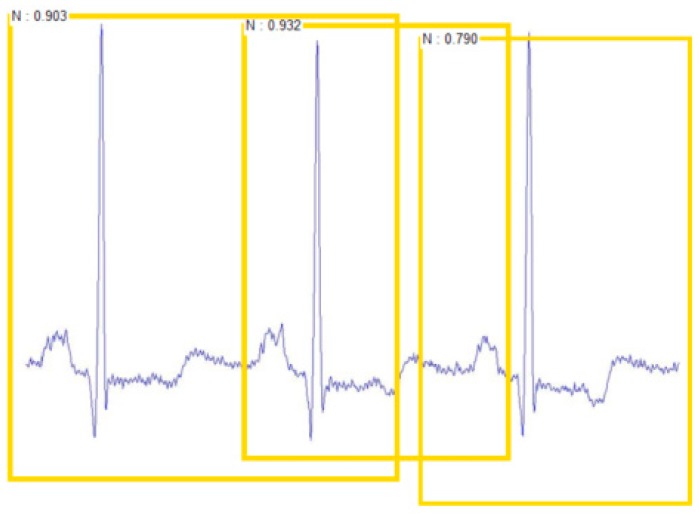
The detected sample graph consisting of multiple ECG beats.

Table 9 shows the classification results of Faster R-CNN. The classification accuracy of each class was more than 99%. The average classification accuracy rate was 99.21%. For testing the robustness of the test classification model, this experiment made a test set with multiple ECG beats in a sample picture, as shown in Figure 14, and still showed good classification results.

Since SVM is also a widely applied classification method in ECG arrhythmia detection, we did a comparative experiment with the OVR SVM method. SVM is a binary classifier, which was originally designed for binary classification problems. When dealing with multi-class problems, it is necessary to construct an appropriate multi-class classifier.

We realized the construction of multiple classifiers by combining multiple binary classifiers. During training, the samples of one category are successively classified into one category, and the rest of the samples are classified into another category. In this paper, we needed to divide the ECG beats into five categories; thus, the selection of the training set was as shown in Table 10.

**Figure 15:**
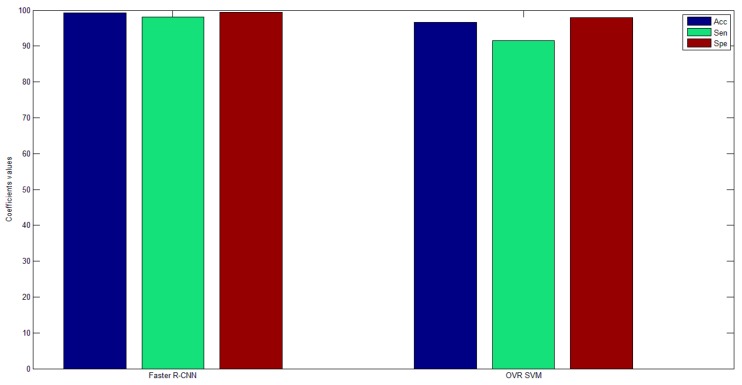
Faster R-CNN and one versus rest support vector machine (OVR SVM) comparison results.

We used these five training sets to obtain five separate training models. Then, we used test sets of each category to test the five training models, whereby each test set got five test results. The largest of the five test results was selected as the final classification result for each test set. Table 11 shows the classification results of the OVR SVM. The average classification accuracy rate was 96.62%. Figure 15 shows the comparison of classification results of the two methods. We can see that the average classification accuracy, sensitivity, and specificity of Faster R-CNN were higher than for OVR SVM.

The benefits of Faster R-CNN are as follows:

- No manual feature extraction is required.
- The sampling rate of the original ECG signal does not need to be considered.
- The approach is insensitive to the ECG signal quality.
- High classification accuracy.

The drawbacks of Faster R-CNN are as follows:

- Training set samples need to be manually labeled.
- Requires long training hours, and specialized hardware to efficiently train datasets (graphics processing unit (GPU)).

However, once the training of the ECG signals is completed, the classification of ECG heartbeat signals is fast. It takes about 0.025 s to classify a test sample of ECG beats.

### 4.2. Comparison with Existing Approaches

Various machine learning methods are also used to classify ECG signals. Therefore, we also compared the classification results of these algorithms. Table 12 presents a performance comparison with previous works. From Table 12, we can see that the proposed method achieved the best results in average accuracy.

## 5. Conclusions

In this paper, we proposed an effective ECG classification method using Faster R-CNN based on a ZF net with ECG images as input. For better-quality ECG images, we used the EMD method to preprocess the one-dimensional ECG signals; then, the DWT algorithm was used to find the R wave position of the ECG signals, and the one-dimensional ECG signals were transformed into two-dimensional ECG images using the sliding window algorithm. After several experiments and parameter optimization, we finally classified the ECG beats into five categories with an average accuracy of 99.21%. Meanwhile, we did a comparative experiment using the OVR SVM algorithm, and the classification result of our method was higher than that of the OVR SVM algorithm. In addition, we also compared with previous works using machine learning algorithms to classify ECG signals, and the proposed method achieved the best results in average accuracy. Furthermore, the proposed ECG classification method can be applied to medical robots. For future work, we will streamline and optimize the model structure of this algorithm so that it can classify ECG signals in real time and play an important role in future healthcare.

## Figures and Tables

**Table 1:** Software information in the experiment.

Software Information	Version Number
MATLAB	2017a
CUDA	8.0
OpenCV	2.4.9
Python	3.5
Visual Studio	2012

**Table 2:** Classification of electrocardiogram (ECG) beats using the AAMI standard [38].

AAMI ECG Beat Class	MIT-BIH ECG Beat Types
Normal (N)	Normal (NOR), left bundle branch block (LBBB), right bundle branch block (RBBB), atrial escape (AE), nodal (junctional) escape beat (NE)
Supraventricular (S)	Atrial premature (AP), aberrated atrial premature (aAP), nodal (junctional) premature (NP), supraventricular premature (SP)
Ventricular (V)	Premature ventricular contraction (PVC), ventricular escape (VE)
Fusion (F)	Fusion of ventricular and normal (FVN)
Unknown (Q)	Paced (/), fusion of paced and normal (FPN), unclassified (U)

**Table 3:** Definition of four classification results.

Type	Definition
True positive (TP)	The number of abnormal ECG beats correctly classified
False positive (FP)	Abnormal ECG beats divided into normal numbers
True negative (TN)	The number of normal ECG beats correctly classified
False negative (FN)	Normal ECG beats divided into abnormal numbers

**Table 4:** Number of ECG beats per class in the MIT-BIH database.

Type	Records	Number
**NOR**	100, 101, 103, 105, 108, 112, 113, 114, 115, 117, 121, 122, 123, 202, 205, 219, 230, 234	75,052
**LBBB**	109, 111, 207, 213	8074
**RBBB**	118, 124, 212, 231	7259
**PVC**	106, 116, 119, 200, 201, 203, 208, 210, 213, 215, 221, 228, 233	7129
**FVN**	108, 109, 114, 124, 200, 201, 202, 203, 205, 208, 210, 213, 214, 215, 219, 223, 233	803

**Table 5:** Number of ECG beats per class used in the experiment.

Type	NOR	LBBB	RBBB	PVC	FVN	Total
Label	N	L	R	V	F	
Patient data	2000	2000	2000	2000	2000	10,000
MIT-BIH data	8500	8074	7259	7129	8030	38,992
Training set number	5250	5037	4630	4565	5015	24,497
Test set number	5250	5037	4629	4564	5015	24,495

**Table 6:** Learning rate test results.

Learning Rate	Loss	mAP
0.0001	0.011	0.992
0.001	0.015	0.987
0.01	0.031	0.952
0.1	0.072	0.85

**Table 7:** Weight attenuation coefficient test results.

Weight Decay	Loss	mAP
0.0001	0.006	0.981
0.0005	0.013	0.987
0.001	0.017	0.983
0.05	0.052	0.974
0.1	0.064	0.970

**Table 8:** Iteration number test results.

Iterations	mAP
2000	0.86
4000	0.987
6000	0.954
8000	0.923

**Table 9:** The classification results of Faster R-CNN. Acc—accuracy; Sen—sensitivity; Spe—specificity.

**Reference Label**		**Algorithm Label**							
	**Type**	**N**	**L**	**R**	**V**	**F**	**Acc (%)**	**Sen (%)**	**Spe (%)**
	**N**	5149	33	30	9	29	99.11	98.27	99.39
	**L**	13	4975	21	4	24	99.32	98.77	99.47
	**R**	35	27	4520	12	35	99.09	97.65	99.43
	**V**	52	15	26	4452	19	99.38	97.54	99.44
	**F**	17	29	37	14	4918	99.17	98.07	99.50

**Table 10:** Train set of the one versus rest support vector machine (OVR SVM).

Type	Positive Sample	Negative Sample
Training set number (N)	5250	19,245
Training set number (L)	5037	19,458
Training set number (R)	4629	19,866
Training set number (V)	4564	19,931
Training set number (F)	5015	19,480

**Table 11:** The classification results of the OVR SVM.

**Reference Label**		**Algorithm Label**							
	**Type**	**N**	**L**	**R**	**V**	**F**	**Acc (%)**	**Sen (%)**	**Spe (%)**
	**N**	4917	67	107	43	116	96.82	93.66	97.69
	**L**	175	4465	120	126	151	96.35	88.64	98.35
	**R**	114	57	4252	114	92	96.55	91.86	97.64
	**V**	82	111	147	4117	107	96.68	90.21	98.16
	**F**	74	87	95	84	4675	96.71	93.22	97.61

**Table 12:** Comparison with existing approaches. 1D—one-dimensional; LSTM—long short-term memory; RNN—recurrent neural network; K-NN—K-nearest neighbor.

Method	Work	Type	Acc (%)	Sen (%)	Spe (%)
**Faster R-CNN**	**Proposed**	**5**	**99.21**	**98.06**	**99.45**
Nine-Layer CNN	U Rajendra Acharya [26]	5	94.03	96.71	91.54
1D CNN	Kiranyaz et al. [21]	5	96.4	68.8	99.5
CNN	Zub air et al. [39]	5	92.70	-	-
CNN + LSTM	Shu Lih Oh et al. [31]	5	98.10	97.50	98.70
RNN	Ubeyli et al. [17]	4	98.06	98.15	97.78
K-NN	Prasad et al. [40]	3	97.65	98.16	98.75
K-NN	Park et al. [19]	17	97	96.6	95.8
